# Do Stainless-Steel Pins Coated with Fibroblast Growth Factor–Calcium Phosphatase Composite Layers Have Anti-Infective Effects?

**DOI:** 10.3390/medicina60091419

**Published:** 2024-08-30

**Authors:** Yasukazu Totoki, Hirotaka Mutsuzaki, Yohei Yanagisawa, Yu Sogo, Mayu Yasunaga, Hiroshi Noguchi, Yukei Matsumoto, Masao Koda, Atsuo Ito, Masashi Yamazaki

**Affiliations:** 1 Department of Orthopaedic Surgery, University of Tsukuba, Tsukuba 305-8571, Ibaraki, Japan; yasutotoki@tsukuba-seikei.jp (Y.T.); yanagisawa@tsukuba-seikei.jp (Y.Y.); noguhiro0164@tsukuba-seikei.jp (H.N.); yukeimatsumoto@tsukuba-seikei.jp (Y.M.); masaokod@tsukuba-seikei.jp (M.K.); masashiy@tsukuba-seikei.jp (M.Y.); 2 Center for Medical Science, Ibaraki Prefectural University of Health Sciences, Ami 300-0394, Ibaraki, Japan; 3 Department of Orthopedic Surgery, Ibaraki Prefectural University of Health Sciences Hospital, Ami 300-0331, Ibaraki, Japan; 4 National Institute of Advanced Industrial Science and Technology (AIST), Health and Medical Research Institute, Tsukuba 305-8566, Ibaraki, Japan; yu-sogou@aist.go.jp (Y.S.); m-yasunaga@aist.go.jp (M.Y.); atsuo-ito@aist.go.jp (A.I.); 5 Department of Orthopaedic Surgery, Ichihara Hospital, Tsukuba 300-3295, Ibaraki, Japan

**Keywords:** bone fixation, calcium phosphate–fibroblast growth factor, external fixation, pin insertion, stainless steel, titanium

## Abstract

*Background:* The most problematic complication of external fixation is infection at the pin insertion site. Technology that improves the adhesion of the external fixation pin to the skin, subcutaneous tissue, and bone may prevent infection at the pin site. The purpose of this study is to formulate a calcium phosphate–fibroblast growth factor (Cp-FGF) coating on a stainless-steel external fixation pin and to verify its effectiveness in reducing infection at the pin site and its possible influence on bone fixation in animal experiments. *Methods:* We compared stainless-steel screws without coating (SS group; *n* = 32), those with a calcium phosphate coating (Cp group; *n* = 30), those with a Cp-FGF coating (FGF group; *n* = 32), and those with a Cp-FGF coating having enhanced biological activity (FGF+ group; *n* = 32) in male Japanese white domesticated rabbits. Screws were inserted percutaneously into the bilateral proximal tibial diaphysis of the rabbits and implanted for 4 weeks. Screws and periscrew tissue were observed postoperatively for qualitatively assessing infection. *Results:* Infection assessment by gross findings after 4 weeks (at screw removal) showed no significant differences between the groups. Histopathological evaluation of soft tissue infection and bone tissue infection showed no significant differences between the groups for either soft tissue or bone tissue. Since neither the FGF+ group nor the FGF group showed anti-infective effects, the biological activity of FGF is not the only determining factor. We compared SEM, XRD, coating detaching test, sustained release test, and bioassay to examine physicochemical properties among the coatings but found no sufficient differences. *Conclusions:* It is suggested that improving the tissue adhesion to and/or biocompatibility of pins is also important to improve the in vivo performance of Cp-FGF-coated external fixation pins.

## 1. Introduction

The most problematic complication of external fixation is pin site infection. External fixation is an essential technique in the treatment of severe trauma of the extremities, including severe trauma with pelvic fractures and open fractures. Pin site infection and associated pin loosening (reduced bone fixation) in external fixation directly affect the outcomes. The incidence of pin site infections in external fixation varies from 3 to 80% [1,2,3,4,5,6,7]. Osteomyelitis in pin puncture site infection is 0–4% [8]. Continuing treatment with external fixation becomes challenging when osteomyelitis develops, and healing of the fracture will be difficult, resulting in long-term treatment and repeat surgery and a decline in the patient’s activities of daily living. Preventing pin site infections in external fixation is an important issue for improving clinical outcomes.

Improvements in surgical procedures [2,5], pin site care [9,10,11], coating with silver, hydroxyapatite (HA), chlorhexidine and HA complex, and iodine [3,12,13,14] have been used to reduce complications of external fixation pins. HA coatings have been studied the most, but it remains unclear whether they reduce the number of clinical infections [15,16].

Technology that improves the adhesion of the external fixation pin to the skin, subcutaneous tissue, and bone may prevent infection at the pin site [17]. External fixation pins are metal implants that penetrate the skin, creating a gap between the skin and around the implant. The interface with the skin must be quickly repaired without leaving a large wound around the pin during insertion. Previous reports have shown that most pin-site infections occur within 12 days in vivo, with one-third occurring within 4 days [18]. Clinically, 50% of external fixation pins had developed infection at 24 days [19]. This indicates that bacteria can enter through the gap during the period of low adherence to the skin and subcutaneous tissue until the wound heals at the time of insertion.

In addition, a fibrous capsule is formed between the external fixation pin and the tissue [20]. Fibrous capsules contribute to pin site infection by reducing tissue adhesion. This indicates that if the adhesion is low, the external fixation pin is constantly subjected to loading and movement, which causes minute movements between the external fixation pin and the tissue with respect to the bone. Pin movement interferes with bone remodeling at the boundary between bone and pin, forming a fibrous capsule and creating instability between the pin and tissue [21]. Instability between the pin and the tissue increases the movement between the pin and the tissue. The shear forces between the pin and the tissue produce a fibrous capsule that does not adhere directly to the pin. Shear forces between the pin and tissue interfere with the direct adherence of the pin to the skin’s soft tissue, thus creating a gap that acts as an access route for external bacteria. In addition, the fibrous capsule has poor blood flow, which reduces its resistance to infection and consequently exacerbates it.

Mutsuzaki et al. [22] fabricated titanium pins coated with fibroblast growth factor (FGF)–calcium phosphate (Cp) composite layers to reduce infection at the pin site. The Cp-FGF composite layer was formed by coprecipitation of FGF with Cp on the titanium surface [23]. The interface between the skin/subcutaneous tissue and the pin was adhered with Sharpey-like fibers, which are considered to prevent crevice infection [18], and improved adhesion strength to the bone was observed [22]. Bone morphogenetic protein-2, FGF-2, and transforming growth factor-β are known as signaling molecules that promote bone formation and soft tissue regeneration [24,25,26,27,28].

Stainless-steel external fixation pins are superior to titanium external fixation pins in terms of strength, variability in the surgical field, rigidity, and price. Stainless-steel screws are superior in breaking strength against titanium screws [29,30,31]. High material stiffness is a prerequisite for treatment with external fixation. In particular, stainless steel is generally used because it is important to withstand shearing forces in the direction of repeated loading.

Sogo et al. [32] reported a method for forming a Cp-FGF coating on stainless-steel (Cp-FGF-SS) external fixation pins. However, no animal studies evaluating the effectiveness of Cp-FGF-SS external fixation pins in reducing puncture site infection have been reported to date. Therefore, the purpose of this study is to formulate a Cp-FGF-SS external fixation pin and to verify its effectiveness in reducing pin site infection and its possible influence on bone–pin interface strength in animal experiments. If we can reveal the effectiveness of Cp-FGF-SS pins, it will have high clinical significance because of its versatility.

## 2. Materials and Methods

### 2.1. Screws Coated with Fibroblast Growth Factor–Calcium Phosphate Composite Layers

A stainless-steel cancellous screw of 3.5 mm diameter and 30 mm length (Synthes, Zurich, Switzerland) was used as the screw for animal experiments. We used titanium cancellous screws and stainless-steel screws in the experiments. All raw materials required for the fabrication of coated screws and drugs and medical devices are approved by the Japanese Ministry of Health, Labour and Welfare and available for use in patients in clinical practice (Table 1). The reagents used in the preparation of supersaturated Cp solutions (soaking solutions) containing FGF are listed in Table 1. Immersion solutions F0 and F4 in Table 2 were prepared using the pharmaceuticals in Table 1.

The animals were divided into five groups: SS group, Cp group, FGF group, and FGF+ group. Stainless-steel screws without coating were used in the SS group, stainless-steel screws with calcium phosphate coating on the surface were used in the Cp group, stainless-steel screws with Cp-FGF coating formed on the surface were used in the FGF group, stainless-steel screws with Cp-FGF coating having enhanced biological activity were used in the FGF+ group (Table 2 and Table 3). Titanium screws coated with Cp-FGF on the surface were fabricated for in vitro study and used in the FGF-Ti group (Table 2 and Table 3).

As shown in Table 3, three stainless-steel screws were immersed in 100 mL of F0 solution at 37 °C for 48 h to coat Cp (Cp group). Three stainless-steel screws were coated with Cp-FGF by immersion in 100 mL of F0 solution for 4 h at 37 °C, followed by immersion in 100 mL of F4 solution for 44 h at 37 °C (FGF group). Stainless-steel screws were coated with Cp-FGF by immersion in 30 mL of F4 solution, to which 1 mL of 100 μg/mL FGF-2 solution was added to increase the FGF-2 concentration to 7.1 μg/mL, and the FGF group stainless-steel screws were immersed in this solution for an additional 12 min (FGF+ group). Controls were uncoated screws (SS group). Three titanium screws were immersed in 100 mL of F4 solution at 37 °C for 48 h and coated with Cp-FGF (FGF-Ti group).

### 2.2. Scanning Electron Microscope of the Coating Layer

To evaluate the screw surface, a scanning electron microscope (SEM; JSM-7400F, JEOL, Tokyo, Japan) was used to compare the surface microscale shape.

### 2.3. X-ray Diffraction of the Coating Layer

The crystallinity and composition of the coatings were compared by X-ray diffraction (XRD). The coating layers of each group—Cp, FGF, FGF+, and FGF-Ti groups—were scraped off with a stainless-steel spatula and used as samples. The samples were placed on a silicon nonreflective plate and measured using a powder X-ray diffractometer (RINT 2550, Rigaku, Japan) with a CuKα beam at 40 kV 200 mA.

### 2.4. Coating Detaching Test

Coating detachment tests were performed using simulated bone. The amount of calcium remaining in the screw after removal from the simulated bone was measured in a simulated bone detachment test. The groups assessed were the Cp group, FGF group, FGF+ group, and FGF-Ti group. Each pin was immersed in 2 mL of 10 mM citrate–citrate buffer (pH 5.43) for 30 min at room temperature to completely dissolve and extract calcium. Extracts were quantitatively analyzed by inductively coupled plasma atomic emission spectrometry (SPS7800; Seiko Instruments Inc., Chiba, Japan). The groups assessed were the SS, Cp, FGF, FGF+, and FGF-Ti groups. The screws were inserted into a simulated bone and a laminated block (SAW1522-11-01P6 CR; Avis, Tokyo, Japan). A 2.8 mm diameter drill (part number 310-250, Synthes, Zurich, Switzerland) was inserted into the simulated bone and laminate and predrilled. The depth of penetration was from the tip of the drill to 2 cm (the most caudal end of the thread). A screwdriver tip (part number 314-030; Synthes, Zurich, Switzerland) was attached to a Jacobs chuck (custom-made chuck for torque measuring instruments by IMADA; IMADA, Toyohashi, Aichi). The measurement method was PEAK using a torque measuring instrument (HTG2-2N; Imada, Aichi, Japan). Screws were inserted and removed from the drill holes. The measured value displayed on the monitor at each insertion and removal was recorded as the maximum insertion and removal torque. The Fixation Index [33] was determined by dividing the removal torque by the insertion torque.

### 2.5. Sustained Release Test

A sustained release test of FGF-2 from the coating was performed. The groups assessed were the Cp-FGF group, FGF+ group, and FGF-Ti group. Screws from each group were rinsed with 1.2 mL of saline and immersed in 1.2 mL of fresh saline at 37 °C (*n* = 6). The saline solution was replaced with a new saline solution daily for 4 days. Subsequently, it was left for 7 days without replacement. The amount of FGF-2 dissolved in saline was analyzed by fluorescence quantification (excitation wavelength: 470 nm, emission wavelength: 570 nm) using the NanoOrange^™^ Protein Quantitation Kit (Thermo Fisher Scientific Inc., Tokyo, Japan) at 1, 2, 3, 4, and 11 min after the start of immersion according to the manufacturer’s instructions. The amount of FGF-2 analyzed was accumulated to create a sustained release curve.

### 2.6. Bioassay

Pins from the FGF, FGF+, and Cp groups were immersed in 2 mL of 10 mM citrate–citrate buffer (pH 5.43) for 30 min at room temperature to completely dissolve and extract the coating.

Cultured mouse fibroblast-derived cells (NIH3T3) (RIKEN Bio-Resource Center, Tsukuba Japan) were treated in Dulbecco’s modified Eagle’s medium (WakoPure Industries, Ltd., Osaka, Japan), to which 10% calf serum (Gibco, Grand Island, NY, USA) was added at 37 °C in humidified air containing 5% CO_2_. The biological activity of FGF-2 was assessed by proliferation analysis using WST-8 reagent (CCK-8; Dojindo Laboratories, Kumamoto, Japan) according to the manufacturer’s instructions and recommendations. The Ca concentration, which affects cell proliferation, was adjusted to equal the lowest concentration of Ca in the extract by dilution with citrate–citrate buffer before analysis (adjusted extract). NIH3T3 was seeded in 96-well plates at a density of 3 × 10^3^ cells per well and starved for 24 h in serum-free medium, to which L-glutamine (0.3 mg/mL) (MP-Bio), bovine serum albumin (1.0 mg/mL) (Gibco), insulin (5.0 μg/mL) (Wako), and transferrin (1.0 μg/mL) (Calbiochem, San Diego, CA, USA) were added. Subsequently, 30 µL of adjusted extract was added to each well. After another 72 h, 10 μL of WST-8 reagent was added to each well. After 2 h, absorbance was measured at 450 nm using a microplate reader (Model 680, Bio-Rad, Hercules, CA, USA). Relative proliferative capacity was defined as the absorbance of the adjusted extract containing FGF-2 relative to the absorbance of the adjusted extract in the CP layer. Relative proliferation values > 1 were considered as retention of the mitogenic activity of FGF-2.

### 2.7. Animal Experiments

This animal experiment was approved by the Ethics Committee for Animal Experiments (Animal Experiment Approval No. 19-180, 1 June 2019).

The SS group, Cp group, FGF group, and FGF+ group were compared. The subjects were 65 (130 limbs) male Japanese white domesticated rabbits weighing 2.5–3.0 kg and aged 10–13 weeks. As reported in a previous study [18], screws were inserted percutaneously into the bilateral proximal tibial diaphysis of a domesticated rabbit and implanted for 4 weeks.

Surgery was performed by subcutaneous injection of ketalar (1 mL/kg) and ceractal (0.7 mL/kg), followed by local anesthesia with 1% xylocaine with epirenamine. A longitudinal skin incision was made on the medial side of the tibia approximately 15 mm distal to the knee joint, dissected to the periosteum, predrilled with a 2.8 mm diameter drill, and tapped with a 3.5 mm diameter, and the screws were inserted bicortically. To measure the maximum torque with a torque meter during insertion, a screwdriver tip (part number 314-030; Synthes, Zurich, Switzerland) was attached to a Jacobs chuck (custom-made chuck specially designed for torque measuring devices by IMADA; IMADA, Toyohashi, Aichi). The measurement method was PEAK using a torque measuring instrument. Screws were inserted into the drill holes, and the maximum insertion torque was measured. To eliminate individual differences, we planned to insert different screws on each side. No randomization was performed. The epidermis was sutured with 4-0 nylon. After surgery, the animals were kept in cages and no specific behavioral restrictions were placed on them.

**Figure 1:**
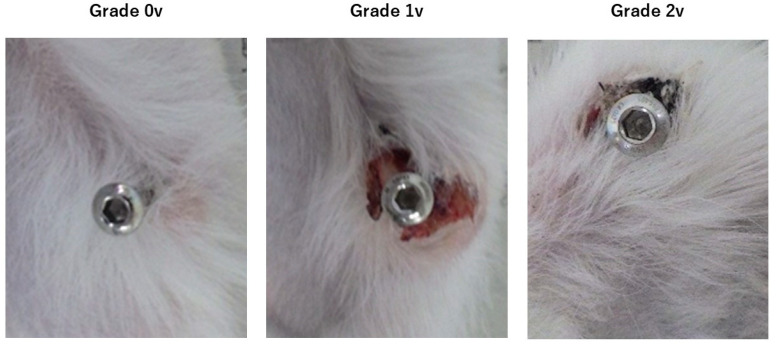
Gross visual findings (screws inserted in and around the tibia of a house rabbit). The background is a Japanese newspaper.

Screws and periscrew tissue were observed postoperatively for qualitative assessment of infection. Specifically, the visual inspection of the soft tissue around the screw and the relationship between the screw and the tibia were evaluated in the following three-step grade classification [18] (Figure 1):

Grade 0v: No skin redness, no leaching, and no loosening of screws.

Grade 1v: Redness of skin present, erosion present, and no loosening of the screw. The criterion for the presence of redness is that the redness extends over an area ≥10 mm around the pin, and infection is assumed to be confined to the soft tissues.

Grade 2v: Redness of skin, erosion, and loosening of the screw are present. Loosening of the screw is a possible osteomyelitis condition.

Grade 1v and 2v were considered as infected. Spontaneous screw dropout during the course of the procedure was considered Grade 2v. Looseness was assessed by touching all the screws. At 4 weeks postoperative, the animals were euthanized by an overdose of anesthetic (Ketalar and Ceractal), the screws were removed, the maximum removal torque was measured, specimens were collected for bacteriological examination, and bone and soft tissue specimens were collected at the screw insertion site. To measure the removal torque, the screws were extracted using a torque-measuring instrument in the same manner as that during insertion, and the maximum measured value displayed on the monitor was recorded as the removal torque. The Fixation Index was determined by dividing the removal torque by the insertion torque to minimize the effects of individual differences. Samples for bacterial examination were collected by inserting a Seed Swab^®^γ No. 2 ‘Eiken’ (E-MS 62; Eiken Chemical Co., Ltd., Tokyo, Japan) into the hole from which the screw was removed, smeared on blood agar medium and egg yolk medium, and incubated for 24 h. Bacteria were identified using media requested from the Central Institute for Experimental Animals (Kanagawa, Japan), on which bacterial growth could be observed macroscopically. To obtain bone and soft tissue specimens at the screw insertion site, the lower leg was disarticulated below the knee and proximal half of the lower leg. The entire soft tissue was fixed in 10% formalin. Soft tissue specimens (tissue samples) from the skin to the periosteum, including the screw holes, were prepared. The proximal diaphysis of the tibia, including the screw hole, was also collected and demineralized to prepare a bone specimen (tissue sample). Those soft tissue and bone specimens were stained using hematoxylin and eosin (HE).

**Figure 2:**
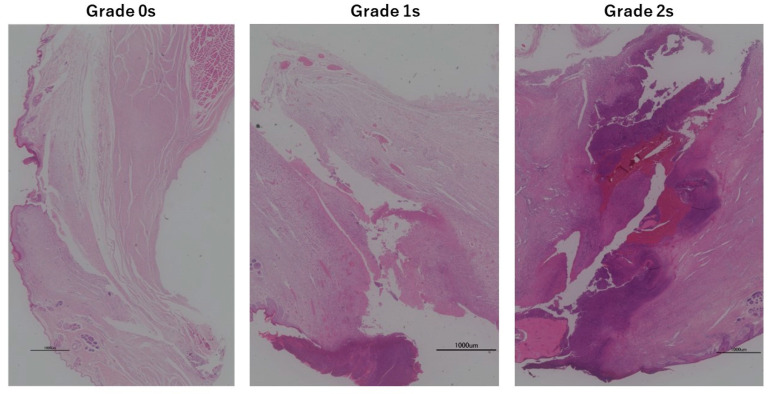
Tissue specimen (soft tissue of tibia, around screw insertion site).

Soft tissue infections were also evaluated histopathologically using soft tissue specimens. That is, the soft tissue specimens were HE-stained, and screw holes reaching from the epidermis to the periosteum were observed in one field of view and classified and evaluated into the following three-step grading [18] (Figure 2):

Grade 0s: No inflammatory findings in the soft tissue around the screw hole.

Grade 1s: Inflammation of some soft tissues around the screw hole.

Grade 2s: Inflammatory findings in the entire length of the soft tissue.

**Figure 3:**
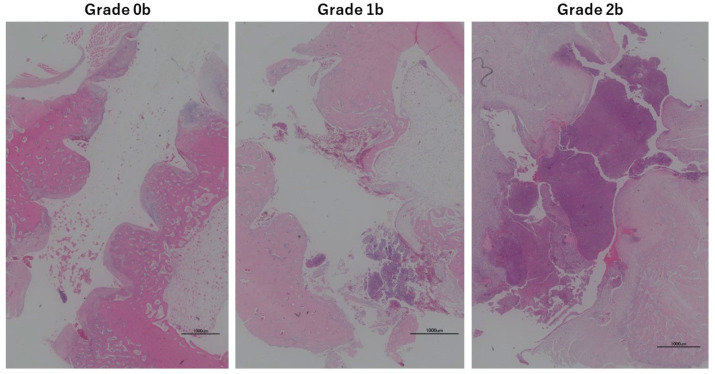
Tissue specimen (bone of tibia, around screw insertion site).

Infection of bone tissue was also assessed histopathologically using bone specimens, i.e., bone specimens were prepared to include the entire length of the screw hole from the cortex to the opposite cortex. Screw holes were observed in one field of view and evaluated in the following three-step grading [18] (Figure 3):

Grade 0b: No inflammatory findings in the bone tissue around the screw hole.

Grade 1b: Inflammation of some of the bone tissue around the screw hole.

Grade 2b: Inflammatory findings are present along the entire length of the screw hole.

### 2.8. Statistical Analysis

SPSS Statistics (IBM^®^ version 28.0.0.0) was used for statistical analysis, and the significance level was set at 5%. Kruskal–Wallis test was used to compare the measured peak torques and sustained release test. Chi-squared test was used to compare the number of infections, and the Kaplan–Meier method was used to create survival curves.

## 3. Results

### 3.1. Scanning Electron Microscope of the Coated Layer

**Figure 4:**
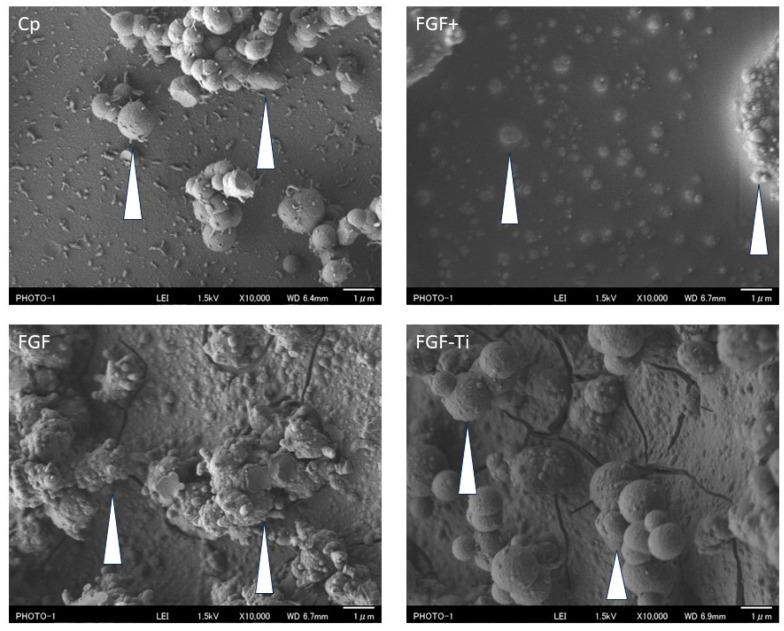
Observation of coated surface using scanning electron microscopy for Cp, FGF, FGF+, and FGF-Ti. Islands of Cp are shown (white arrows).

**Figure 5:**
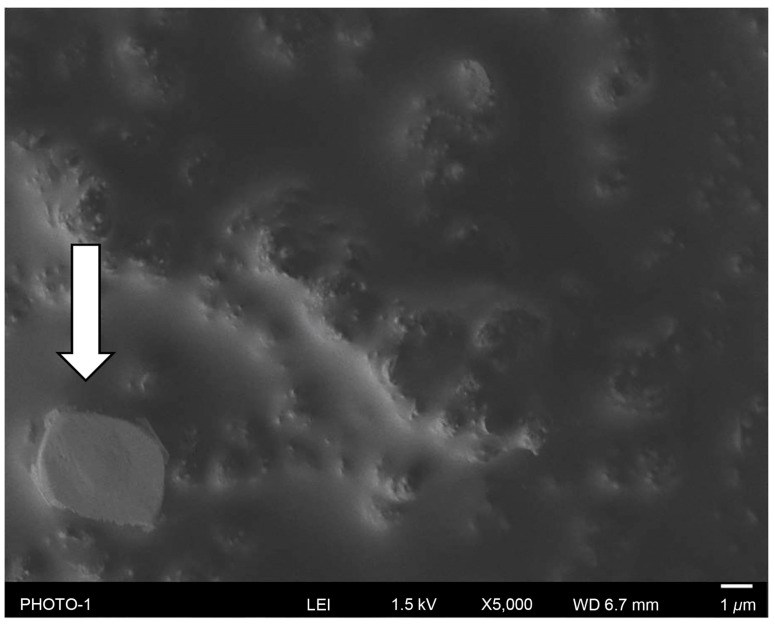
Crystal structure attached to the surface of FGF+ (white arrow).

SEM showed that the metal surfaces of the Cp, FGF, FGF+, and FGF-Ti groups were all coated with Cp. In addition, 1–10 µm–large islands of Cp were observed adhering to the surface (Figure 4; Cp islands are indicated by arrows). Cracks were observed on the surface of the coating in the FGF and FGF-Ti groups. In FGF+, the space between the large Cp islands appears uniform. Small Cp islands are observed between black uniform surfaces. Square crystal-like structure observed only in FGF+ (Figure 5; crystal is indicated by arrow).

### 3.2. X-ray Diffraction of the Coated Layer

**Figure 6:**
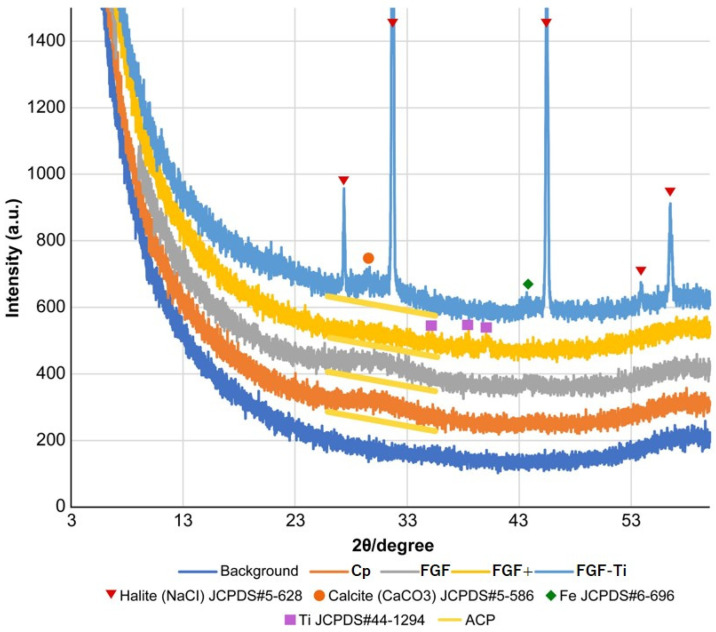
X-ray diffraction patterns for Cp, FGF, FGF+, FGF-Ti and background.

XRD showed a peak-like increase in X-ray intensity at 25°–33° and centered around 30° in all specimens, indicating the presence of amorphous calcium phosphate (ACP) [34]. The stainless-steel component Fe was detected in the FGF and FGF+ groups, and the Ti peak was detected in the FGF-Ti group. Multiple strong peaks present only in the FGF+ group were NaCl precipitated by drying of additional soaking solution (Figure 6). Because NaCl dissolves and disappears in body fluids, the composition remaining during implantation is ACP for both the FGF, the FGF+, and the FGF-Ti. There was no difference in the crystallinity of these ACPs.

### 3.3. Coating Detachment Test

**Figure 7:**
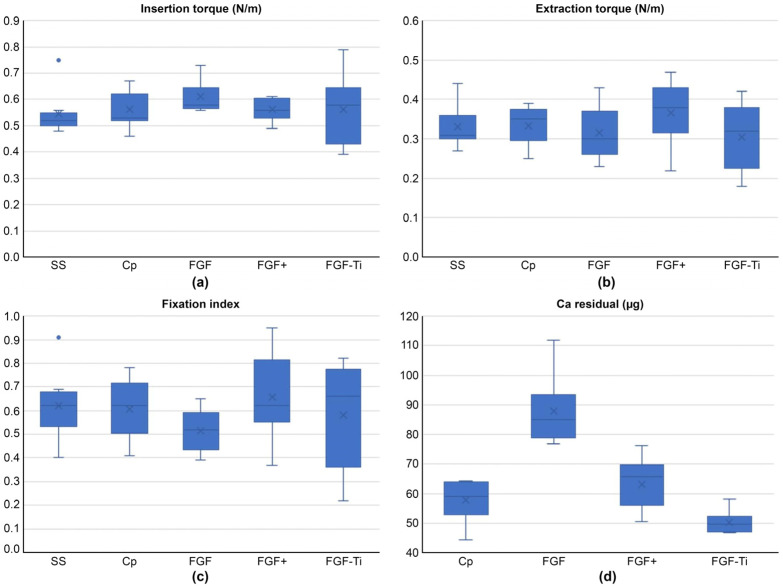
Box-and-whisker plots for experimental measurements of coating detaching test. (**a**) is Insertion torque; (**b**) is removal torque; (**c**) is Fixation Index of SS, Cp, FGF, FGF+, and FGF-Ti; (**d**) is the amount of Ca residual of Cp, FGF, FGF+, and FGF-Ti. Dots in (**a**,**c**) are outliers.

No difference in insertion or removal torque was observed between the FGF, FGF+, and FGF-Ti groups. The Fixation Index was also comparable among the FGF, FGF+, and FGF-Ti groups (Figure 7). In other words, no significant difference in anti-loosening performance because of frictional resistance was observed between the Cp-FGF coating on the stainless-steel screw and the Cp-FGF coating on the titanium screw.

Ca supported on the screw was 131.8 ± 23.7 μg in the Cp group, 170.4 ± 47.6 μg in the FGF group, 152.8 ± 16.5 μg in the FGF+ group, and 143.5 ± 60.9 μg in the FGF-Ti group [35]. Ca remained on the screw, but there was a significant difference in the amount of remnant Ca (Figure 7, *p* < 0.001). When comparing between groups, the results were significantly higher in the FGF group than in the Cp group and FGF-Ti group (*p* < 0.001, *p* < 0.001).

### 3.4. Sustained Release Test

**Figure 8:**
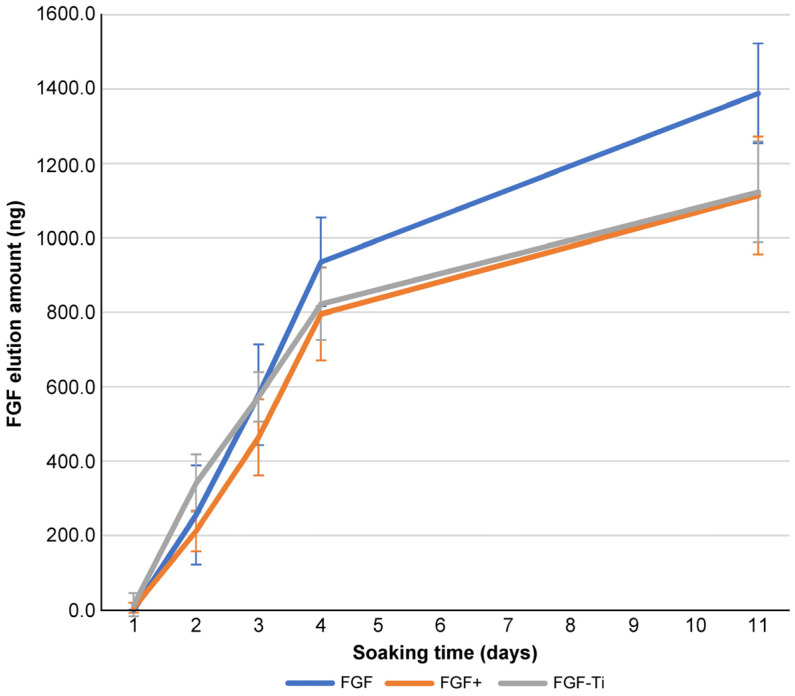
Sustained release test of FGF, FGF+, and FGF-Ti.

The sustained release rates were similar for the FGF+ group and the FGF-Ti group (*p* > 0.05). The FGF group showed a significantly faster elution rate than the FGF+ group on day 3 (*p* = 0.005). The FGF group showed significantly faster elution rates than the FGF-Ti group on days 3, 4, and 11 (*p* = 0.015, *p* = 0.021). The amount of eluted FGF showed no significant differences (Figure 8).

### 3.5. Bioassay

**Figure 9:**
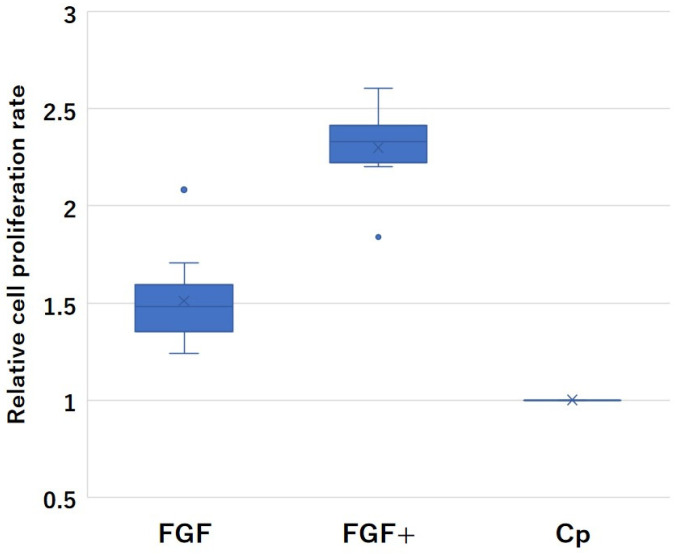
Box-and-whisker plots for relative cell proliferation rates of FGF and FGF+. The dots are outliers. Cp is shown as the reference value of 1 that means threshold of retention of the mitogenic activity of FGF-2.

Relative cell proliferation rates in the bioassay are shown in Figure 9. The cell relative proliferation rates of the FGF and FGF+ groups were 1.51 ± 0.20 and 1.93 ± 0.21, respectively, which were higher than that of 1. In addition, the relative proliferation rate of the FGF+ group had a significantly higher relative proliferation rate than that of FGF (*p* < 0.001).

### 3.6. Animal Experiments

Two rabbits died from anesthesia during surgery; thus, screws were inserted in 32 limbs of the SS group, 32 limbs of the Cp group, 30 limbs of the FGF group, and 32 limbs of the FGF+ group. None of the animals died during the 4-week follow-up.

**Figure 10:**
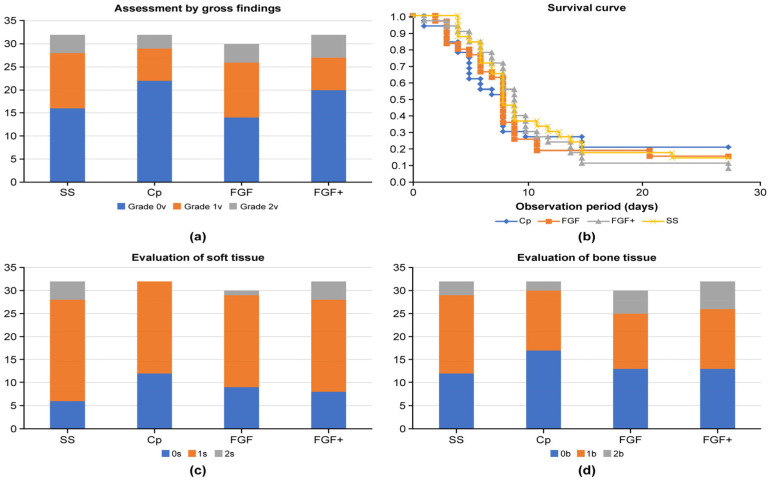
Experimental measurements of animal infections. (**a**) is result of gross findings; (**b**) is survival curve; (**c**,**d**) are from tissue specimens.

The results of infection assessment by gross findings after 4 weeks (at screw removal) are shown in Figure 10a. The screw dropped out in one limb in each of the FGF and FGF+ groups and was determined to be Grade 2v. A Chi-squared test showed no significant difference between the groups. Kaplan–Meier method was used to examine the survival curve (Figure 10b), with the turning point (assumed to be death) being the onset of gross signs of infection. No significant differences were found among the four groups.

Representative histological images with H&E staining for soft tissue and bone are shown in Figure 2 and Figure 3. The results of the evaluation of soft tissue infection and bone tissue infection by histopathological observation are shown in Figure 10c,d. No formation of Sharpey-like fibers reported by Mutsuzaki [18] was observed in the pathology of the soft type classified as grade 0s. At the interface with the screw, fibrous tissue, single cell layer, or neovascularization were not observed. A Chi-squared test showed no significant difference between the groups for either soft tissue or bone tissue.

**Figure 11:**
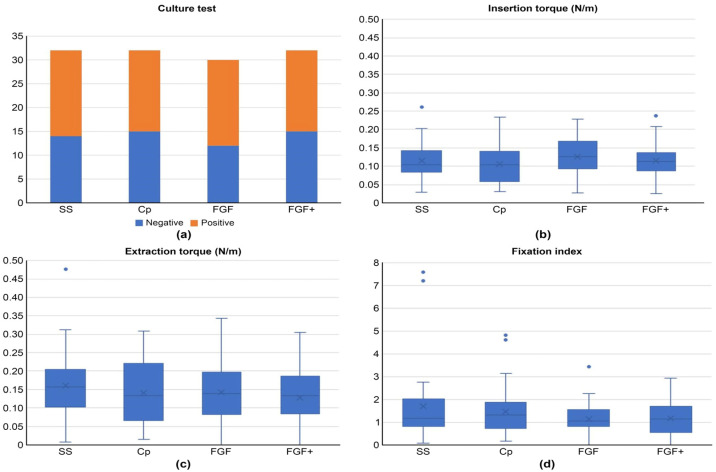
Objective findings of animal experiments. (**a**) is culture test; (**b**–**d**) are box-and-whisker plots for torque measurements of pins, and the dots are outliers.

The results of the bacterial culture are shown in Figure 11a. The bacteria detected were *Staphylococcus aureus* in 66 samples, *Staphylococcus microti* in 4 samples, *Staphylococcus cohnii* in 1 sample, *Enterobacter cloacae* in 1 sample, *Enterobacter bugandensis* in 3 samples, *Escherichia coli* in 1 sample, and *Corynebacterium amycolatum* in 3 samples. A Chi-squared test was performed on the number of samples in which bacteria were detected, and no significant differences were found between the groups.

The insertion torque, extraction torque, and Fixation Index are shown in Figure 11b–d. No statistically significant differences were found in the measured torque values or Fixation Index.

## 4. Discussion

In this study, Cp group, FGF group, and FGF+ group coatings were successfully applied on stainless-steel screws as on the titanium screws in our previous studies. An exploratory clinical study on Cp-FGF-coated titanium external fixation pins [35] successfully demonstrated its safety but indicated the potential for improvement in efficacy in the prevention of puncture site infection. Therefore, we explored various manufacturing methods to enhance the biological activity of Cp-FGF coatings (Figures S1–S3). Based on the results of the search, FGF group and FGF+ group were coated on stainless-steel screws. The cell proliferation rate, a measure of the biological activity of the FGF group, was 1.51 ± 0.20, and the cell proliferation rate of the FGF+ group was 1.93 ± 0.21. These values are in the range of previously reported relative growth rates of Cp-FGF coated on titanium screws (1.3–2.2) [22,32,35,36]. SEM and XRD results showed no difference between FGF and FGF-Ti and differed from the infection results. Similarly, in the sustained release test, FGF+ and FGF-Ti were similar and the infection results were different. Surface structure and sustained release rate did not affect the anti-infection effect.

Contrary to our expectations, there was no significant difference in infection prevention efficacy and bone fixation strength between the SS group, FGF group, and FGF+ group in vivo. The results were in contrast to our previous studies in which Cp-FGF coating was applied to titanium screws. Mutsuzaki et al. [18] reported that the Cp-FGF coating applied to titanium screws reduced the infection rate and bone fixation strength compared with Cp-coating without FGF-2. In the case of FGF-Ti, more than 70% of the coating remained when it penetrated the cortical bone (Table S1). After 4 weeks of implantation in vivo, the coating disappeared in the area that was in contact with the bone but remained on the surface of the soft tissue [37]. The anti-infective effect and bone fixation were different when Cp-FGF coating was applied to stainless-steel screws versus titanium screws. Two hypotheses were considered as possible causes of this discrepancy in anti-infection effects and bone fixation strength of Cp-FGF coating between titanium screws and stainless screws. The first hypothesis is that there is a difference in the physical and/or chemical properties of the Cp-FGF coating on the stainless-steel screw and the titanium screw, resulting in different anti-infective effects and bone fixation properties between the two types of screws. However, this hypothesis may not have a significant influence because there were no significant differences between both Cp-FGF coating screws in terms of composition, microscale shape, crystal structure, anti-exfoliation performance (Figure S4), and FGF sustained release performance.

The second hypothesis is that there is a difference in tissue adhesion to stainless-steel screws and to titanium screws and that this difference results in different anti-infection effects and bone fixation between the two types of screws. Regarding tissue adhesion to the titanium screw, some Sharpey fiber-like tissue was observed in the soft tissue portion in contact with the screw [18]. Similarly, hemidesmosomes and basement membrane components have been observed in the gingival epithelium in contact with titanium and contribute to anti-infection [38,39,40,41]. In the case of the stainless-steel screw, FGF-2 may have caused tissue to fill the gap between the coating but may not provide a firm adherence between the tissue and the stainless-steel screw, from which infection may have occurred. Our study also showed differences in bone matrix-like tissue formation and adhesion between titanium and stainless-steel screws in vitro (Figure S5 and Table S2).

Not fibrous tissue, but normal soft tissue regeneration is necessary for soft tissue adhesion to the screw. Inflammation to the damaged tissue and its suppression work overtime is important to obtain normal soft tissue regeneration. Evaluation of inflammatory cytokines around the metal implanted in rats showed that Ti alloy (Ti6Al4V) suppressed the development of inflammation compared to stainless steel [42]. Increased regulatory T cells control destructive immune responses by preventing the generation of immune responses to self-antigens and harmless external antigens. The increase in regulatory T cells in Ti alloys compared with that in stainless steel may have resulted in increased affinity for autoimmunity and caused immune tolerance. In contrast, stainless steel increased the tendency for chronic inflammation and apoptosis [43]. Sustained proinflammatory responses promote fibrosis through the IL-17A–IL-1b–TGF-b1 axis [44].

In animal studies [45], angiogenesis was observed only with Ti. The oxide film that forms on the Ti surface is reported to protect microcirculation, with the result that Ti has infinitely less hemodynamic impact (i.e., inflammation) on the surrounding tissues than stainless steel or silver. Thus, it is suggested that the action of FGF-2 on mesenchymal stem cells in an environment with a suppressed inflammatory response enhances the formation of normal tissue, including blood vessels, on titanium but fibrous connective tissue on stainless steel. To prevent wound site infection of external fixation pins with Cp-FGF coating, it may be important to inhibit the induction of inflammatory cytokines by the substrate to which the Cp-FGF coating is applied and to improve the biocompatibility of the substrate.

Limitations of this study are that the animal studies were conducted over a short period of time (4 weeks) and that no direct comparison was made between titanium and stainless-steel screws. The insertion and removal torque are determined by three factors: the adhesion between the screw and the coating, the strength of the coating, and the adhesion between the coating and the bone. Because titanium screws were not used in animal experiments, the difference in torque due to metals cannot be evaluated. With these values, another assessment could be made as to whether or not this factor is a determinant.

## 5. Conclusions

Cp-FGF coatings and Cp-FGF coatings having enhanced biological activity on stainless-steel screws were prepared. Neither the FGF+ group nor the FGF group showed anti-infective effects in domesticated rabbits. This study indicates that the in vivo performance of Cp-FGF implants is not only determined by the bioactivity of FGF as a drug but also depends on the tissue adhesion/biocompatibility of the tissue scaffold substrate. To improve the in vivo performance of Cp-FGF-coated external fixation pins, it is necessary to improve the tissue adhesion/biocompatibility of the pins themselves.

## Figures and Tables

**Table 1:** Reagents used to prepare supersaturated calcium phosphate solutions (soaking solutions).

Infusion Solution Containing Calcium
Ringer’s solution, 500 mL (Otsuka, Otsuka Pharmaceutical Factory, Tokushima, Japan)
Calcium chloride correction solution, 1 mEq/mL, 20 mL (Otsuka Pharmaceutical Factory, Tokushima, Japan)
Intravenous solution containing phosphoric acid
Klinisalz infusion solution, 500 mL (Kyowa CritiCare, Tokyo, Japan)
Dipotassium phosphate injection, 20 mEq Kit, 20 mL (Terumo, Tokyo, Japan)
Alkalizing agent (sodium bicarbonate solution)
Meylon Intravenous Injection 7%, 20 mL (Otsuka Pharmaceutical Factory, Tokushima, Japan)
Dissolving and diluting solution
Otsuka Physiological Saline Injection, 20 mL (Otsuka Pharmaceutical Factory, Tokushima, Japan)
Water for injection PL, 20 mL (Fuso, Fuso Pharmaceutical Industries, Osaka, Japan)
FGF-2 (trafermin)
Fiblast Spray 500 (Kaken Pharmaceuticals, Tokyo, Japan)

**Table 2:** Conventional calcium phosphate supersaturated solution.

Name of Soaking Solution	F0	F4
FGF-2 (μg/mL)	0	4
Na^+^ (mM)	138–139	138–139
K^+^ (mM)	6.1–7.4	6.1–7.4
Ca^2+^ (mM)	3.7–4.9	3.7–4.9
Mg^2+^ (mM)	0.2	0.2
Cl^−^ (mM)	134–137	134–137
PO_4_^3−^ (mM)	1.3–1.8	1.3–1.8
HCO_3_^−^ (mM)	15.1	15.1
Temperature (°C)	37	37

**Table 3:** Conventional coating methods.

Name	Substrate	Technique	Components of Coating Layer
Cp-FGF titanium method	Titanium	Immersed in F4 solution for 48 h	Calcium phosphate + FGF-2
Cp-FGF stainless-steel method	Stainless steel	Immersed in liquid F0 for 4 h, then immersed in liquid F4 for 44 h	Calcium phosphate + FGF-2
CP method	Stainless steel, titanium	Immersed in F0 solution for 48 h	calcium phosphate

## Data Availability

The datasets generated and/or analyzed during the current study are available from the corresponding authors upon reasonable request.

## Supplementary Materials

The following supporting information can be downloaded at https://www.mdpi.com/article/10.3390/medicina60091419/s1, Figure S1: Relative cell proliferation rates for FGF concentration; Figure S2: Relative cell proliferation rates for changes in pH; Figure S3: Relative cell proliferation rates for additional immersion; Figure S4: The peeling test with scotch tape; Figure S5: Findings of bone matrix formation study on stainless and titanium screws; Table S1: Intra-bone delivery of Cp-FGF coating; Table S2: The number of bone matrix formation study on stainless and titanium screws.
