# Supplementary material for: Do Stainless-Steel Pins Coated with Fibroblast Growth Factor–Calcium Phosphatase Composite Layers Have Anti-Infective Effects?
Source: Medicina (Kaunas). 2024 Aug 30;60(9):1419. doi: 10.3390/medicina60091419 (PMC11434512; doi:10.3390/medicina60091419)
Supplement: Supplementary file 1 [file medicina-60-01419-s001.zip › medicina-3132470-supplementary.pdf]

Supplementary materials

Do stainless-steel pins coated with fibroblast growth factor–calcium phosphatase composite layers have anti-infective effects?

Yasukazu Totoki [1], Hirotaka Mutsuzaki [2*], Yohei Yanagisawa [1], Yu Sogo [3], Mayu Yasunaga [3], Hiroshi Noguchi [1], Yukei Matsumoto [1], Masao Koda [1], Atuso Ito [3], and Masashi Yamazaki [1]

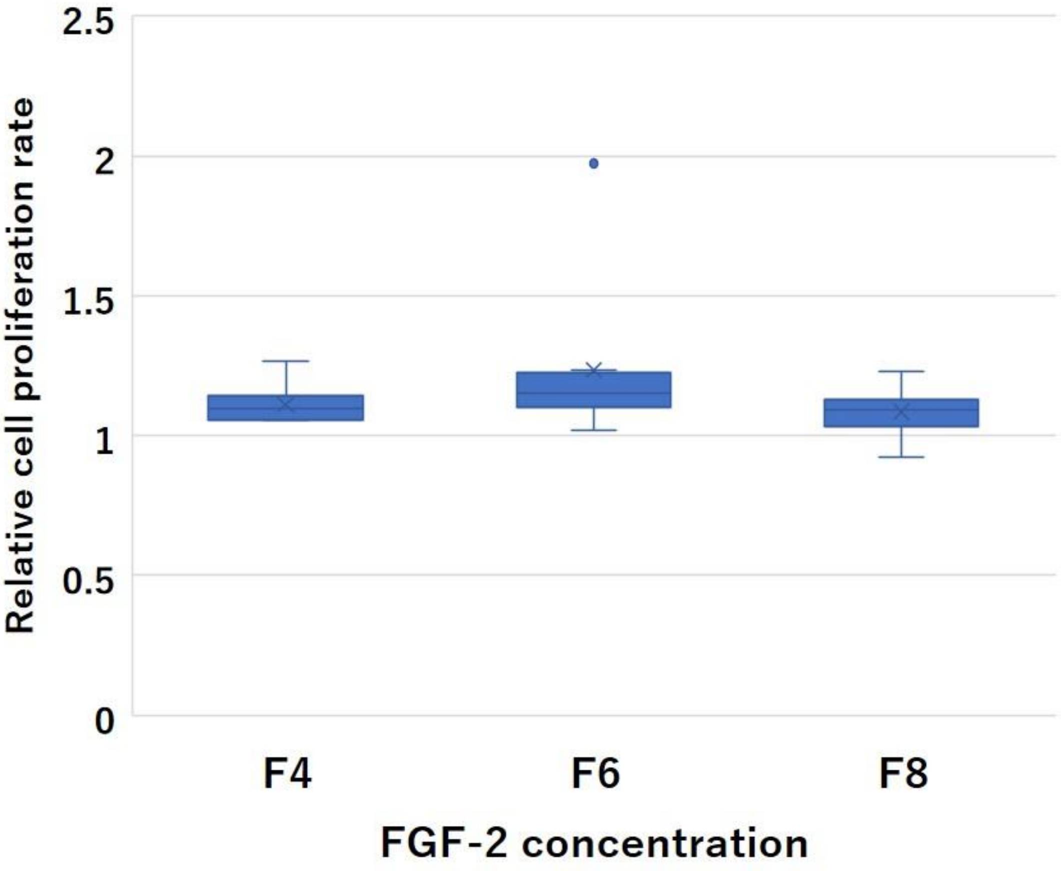

Figure S1. Relative cell proliferation rates for the Cp-FGF stainless-steel screws prepared in the soaking solutions containing 4 (F4), 6 (F6), and 8 (F8) μg/mL of FGF-2 at pH 8.80–8.82. Increases in FGF-2 concentration of soaking solution have no effect on enhancing the biological activity of Cp-FGF coatings.

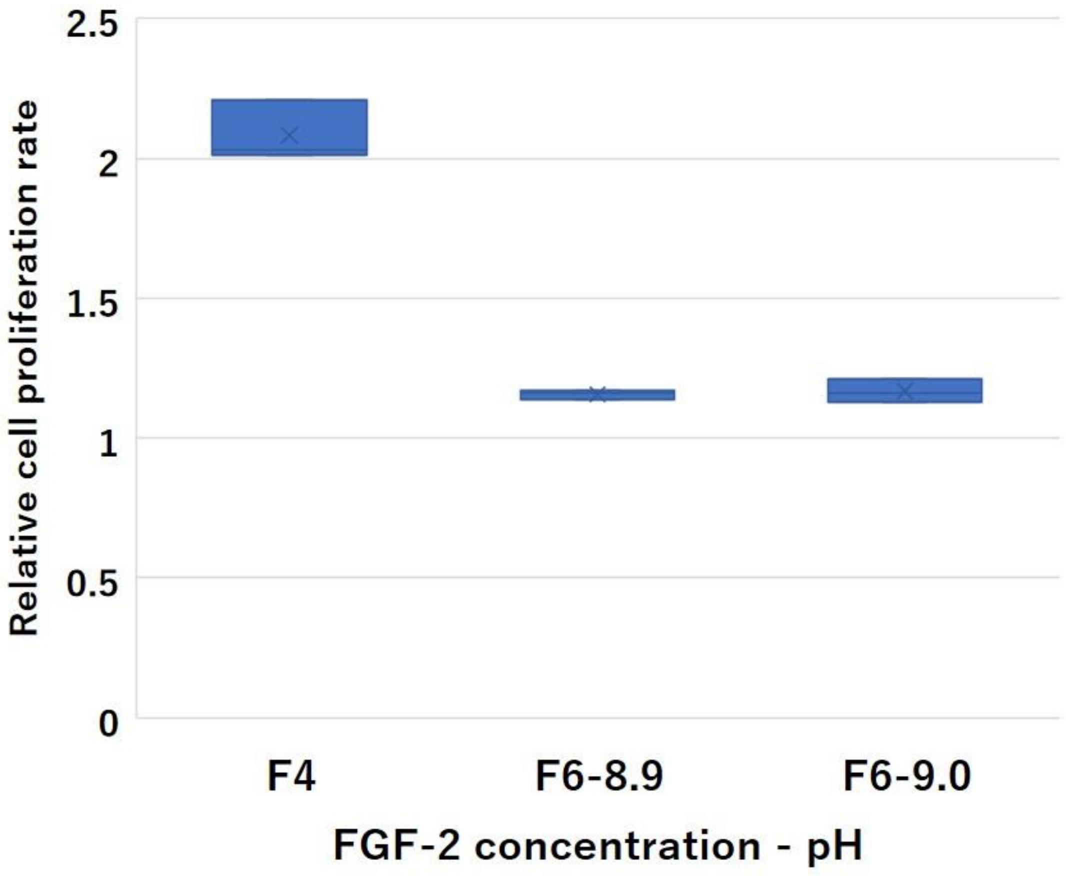

Figure S2. Relative cell proliferation rates for the Cp-FGF stainless steel screws prepared in the soaking solutions at different pH values and FGF-2 concentrations. Increases in pH of soaking solution have no effect on enhancing the biological activity of Cp-FGF coatings.

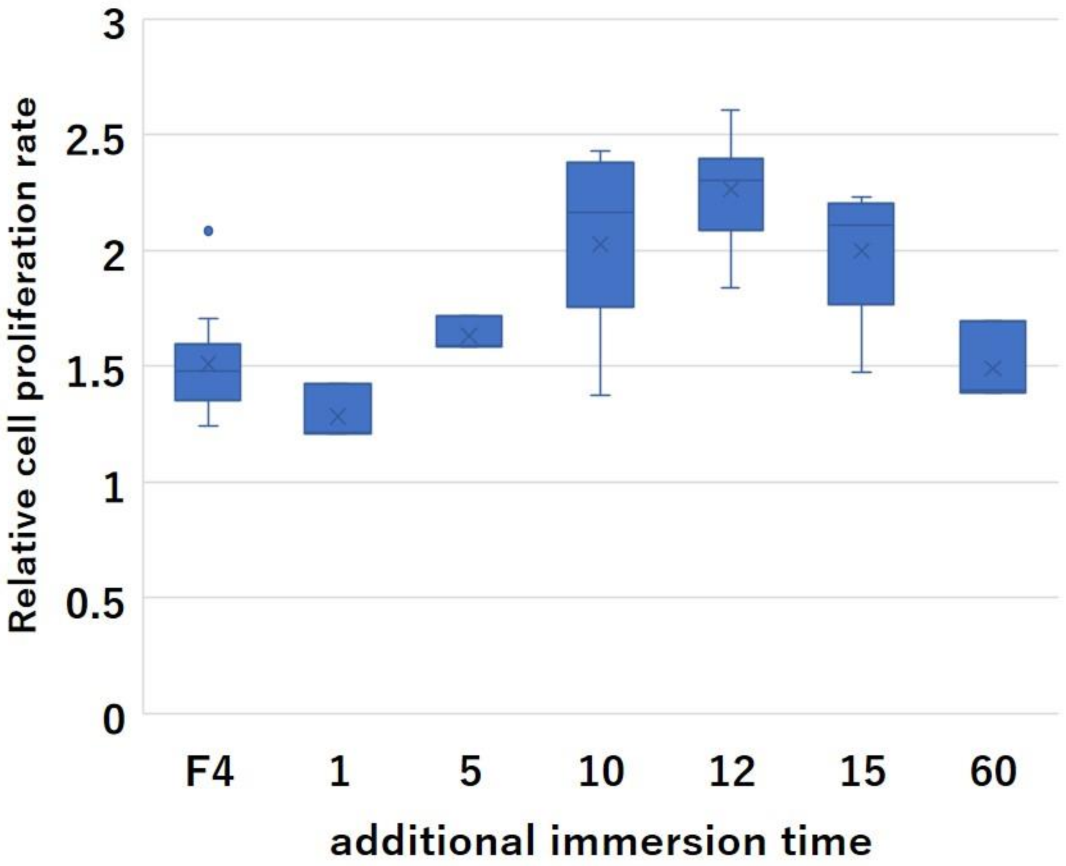

Figure S3. Relative cell proliferation rates for the Cp-FGF stainless steel screws prepared by immersion in the F4 solution for 44 h followed by additional immersion in a F7.1 solution for 1–60 min. Additional immersion in the F7.1 solution for 10, 12, and 15 min have significant effect on enhancing the biological activity of Cp-FGF coatings compared with that of F4 ($p = 0.001$, $p < 0.001$, $p = 0.005$).

In this study, the FGF+ group corresponds to the immersion in the F4 solution for 44 h followed by additional immersion in a F7.1 for 12 min.

Table S1. The amount of coating residual during the process of the screw penetrating the cortical bone was measured. The amount of Ca present in the coating layer was compared before and after penetration through the proximal tibial diaphysis of a Japanese white rabbit cadavers.

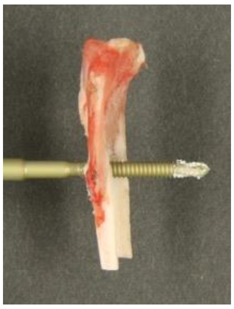

For this test, we used half pins (3.0 mm diameter, 80 mm total length, Pure Ti, Synthes, Zurich, Switzerland) for external fixation. Half pins with Cp-FGF (Control group, n=3) were measured as pre-penetration. Half pins with Cp-FGF (FGF-HP group, n=7) and without Co-FGF (HP group, n=7) were used to penetrate the bone and the amount of Ca after penetration was measured.

The adhesion on the half pins was eluted with citric acid solution, and the amount of Ca was quantified using inductively coupled plasma analysis. The following equation was used to determine the residual rate of Ca.

Residual rate = (FGF – HP) / Control

	Ca residual (μg)
FGF-HP	72.5 ±6.3
HP	21.2 ±5.7
Control	65.4 ±4.9
Residual rate	78.50%

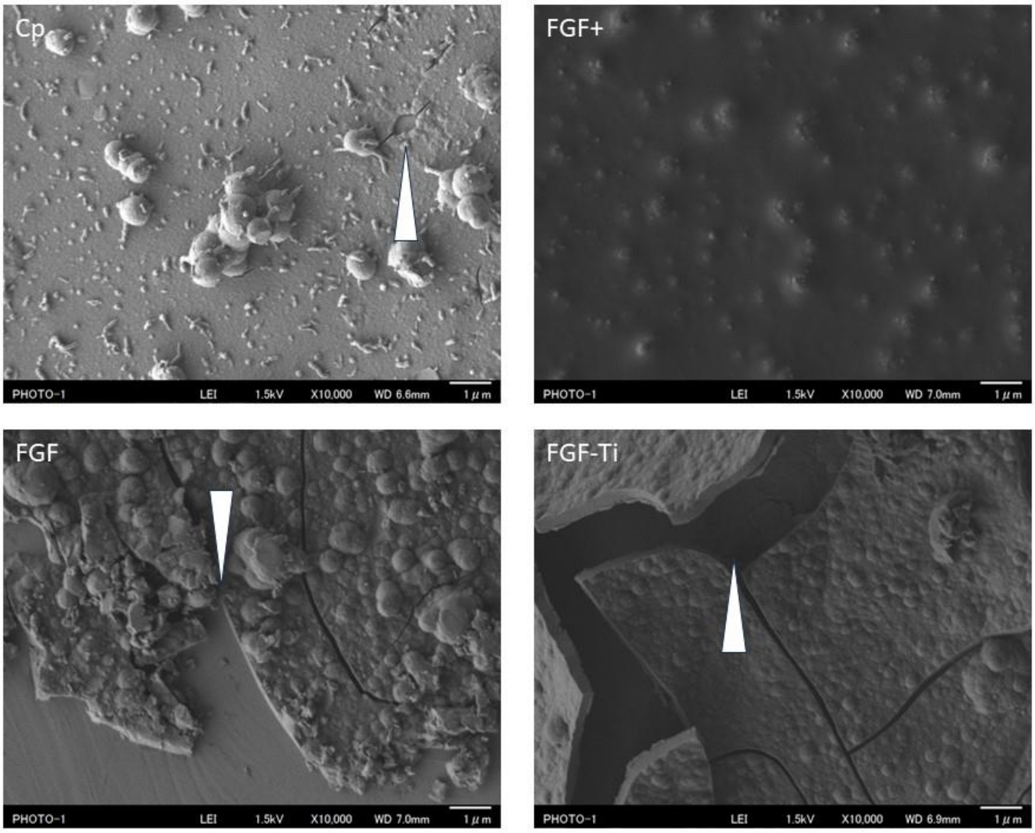

Figure S4. The peeling test with scotch tape was used during surface observation by SEM. The subjects were the Cp group, FGF group, FGF+ group, and FGF-Ti group. The coating was peeled off using Scotch tape (3M, Tokyo, Japan), and the coating surface was observed at the site. It was observed that the coating remained in all cases, although it was partially peeled off (indicated by arrow).

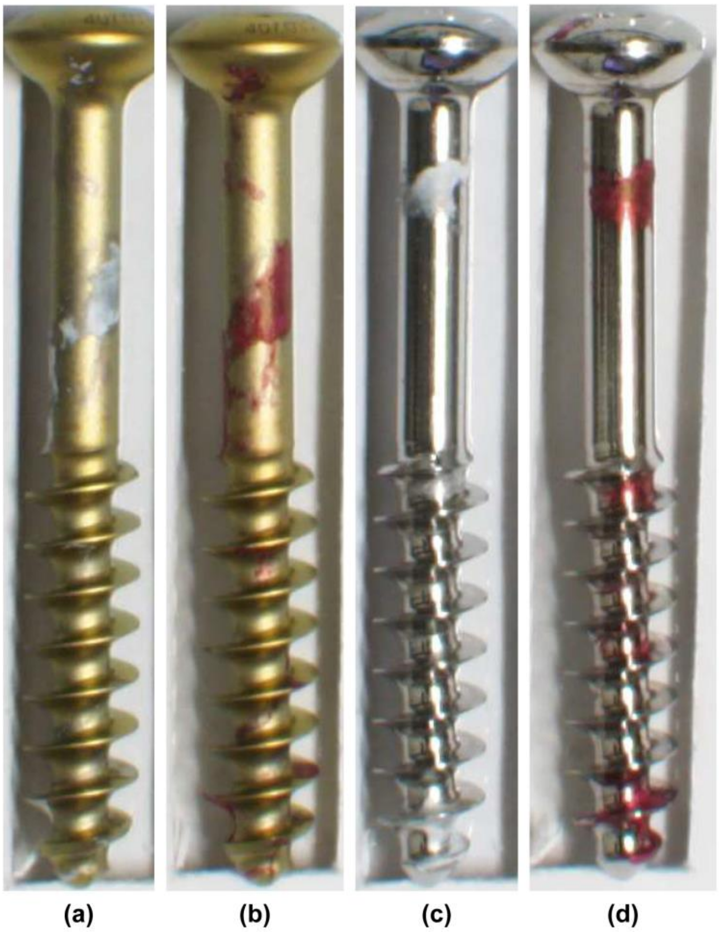

Figure S5. Selected titanium (a, b) and stainless-steel (c, d) screws subjected to in vitro bone matrix formation study with (b, d) and without (a, c) alizarinred s (ARS) staining. Rat mesenchymal stem cells were obtained and cultured on a titanium or stainless-steel screw in a well of 6-well plates at a density of 3×10^5 cells per well under the osteogenic differentiation condition for 4 (first run) or 5 weeks (second run) using a method described elsewhere [42]. Eight titanium and stainless steel screws were used in each experimental run. The medium was renewed three (first run) or two (second run) times a week. After the culture, cells on the screws were lysed with 0.1% Triton X-100 in PBS and frozen for 30 min at −80 °C. After the cell lysis, the screws were dried at room temperature (a, c). Then the screws were dipped for 2 min in 1% alizarinred S (ARS) solution (MUTO PUER CHEMICALS Co., LTD, Tokyo, Japan) at pH 6.3 to stain bone matrix adhered to the screws.

Table S2. Number of ARS-stained screws after the in vitro bone matrix formation study. The number of ARS-stained screw is significantly higher in the titanium group than that in SUS group.

	Number of ARS-stained screws		*p* value
	Titanium	SUS	
Experimental run			
First run	6/8	2/8	0.045
Second run	8/8	4/8	0.020
Total	14/16	6/16	0.003
